# The Biological Effect of Platelet-Rich Plasma on Subacromial Bursa and Torn Supraspinatus Tendon: A Randomized Controlled Trial

**DOI:** 10.3390/ijms27073002

**Published:** 2026-03-26

**Authors:** Charalampos Pitsilos, Aikaterini Fragou, Sofia Karachrysafi, Ioannis Gigis, Konstantinos Ditsios, Byron Chalidis

**Affiliations:** 1 2nd Orthopaedic Department, Aristotle University of Thessaloniki, 54635 Thessaloniki, Greece; chpitsilos@outlook.com (C.P.); jgigis71@gmail.com (I.G.); ditsiosk@otenet.gr (K.D.); 2 Laboratory of Biological Chemistry, Medical Department, School of Health Science, Aristotle University of Thessaloniki, 54124 Thessaloniki, Greece; katerinafragou@hotmail.com; 3 Research Team “Histologistas”, Interinstitutional Postgraduate Program “Health and Environmental Factors”, Department of Medicine, Faculty of Health Sciences, Aristotle University of Thessaloniki, 54124 Thessaloniki, Greece; sofia_karachrysafi@outlook.com; 4 Laboratory of Histology-Embryology, Department of Medicine, Faculty of Health Sciences, Aristotle University of Thessaloniki, 54124 Thessaloniki, Greece; 5 1st Orthopaedic Department, Aristotle University of Thessaloniki, 57010 Thessaloniki, Greece

**Keywords:** platelet-rich plasma, PRP, subacromial bursa, supraspinatus tendon, rotator cuff, tenocyte, collagen

## Abstract

The in vivo effect of platelet-rich plasma (PRP) on supraspinatus tendon morphology and subacromial bursa cell gene expression in degenerative rotator cuff tears remains unclear. This randomized controlled trial evaluated the effect of preoperative leukocyte-poor PRP (LP-PRP) subacromial injection on supraspinatus tendon histology and subacromial bursa gene expression. Sixteen patients with full-thickness supraspinatus tears were randomized to receive an ultrasound-guided LP-PRP injection (*n* = 8) or no injection (*n* = 8) six weeks before arthroscopic repair. Tendon biopsies were assessed using the modified Movin score. Gene expression of collagen type I, II and III, metalloproteinase 3 and 13, and interleukin 1β and 6 genes from subacromial bursa cells was quantified using quantitative real-time PCR. The results of the two groups were compared to determine any statistically significant difference regarding all the examined parameters. The PRP group demonstrated a significantly lower total modified Movin score than controls (6.5 vs. 12.1, *p* = 0.002), with lower scores for fiber structure, fiber arrangement, nuclear rounding, inflammation and cell density (all *p* < 0.003), while angiogenesis did not differ (*p* = 0.149), indicating an architecture closer to that of normal tendon. No statistically significant differences in gene expression were observed (all *p* > 0.05), although collagen II and metalloproteinase 3 and 13 showed biologically relevant downregulation [fold change 0.23 (95%CI 0.05–1.09), 0.24 (95%CI 0.002–26.10), and 0.26 (95%CI 0.02–2.76), respectively]. The LP-PRP injection was associated with improved supraspinatus tendon histological characteristics and biologically relevant reductions in selected bursal genes, in the setting of supraspinatus tendon tear.

## 1. Introduction

The rotator cuff (RC) of the shoulder consists of the subscapularis muscle anteriorly and the supraspinatus, infraspinatus and teres minor muscles posteriorly [1]. The subscapularis is inserted into the lesser tuberosity, whereas the other three are inserted into the greater tuberosity [2]. The supraspinatus tendon lies on the most superior aspect of the RC, and its insertion is formed by the interface of multiple structures [3]. Based on this, it is divided into five layers: 1. superficial fibers of the coracohumeral ligament; 2. dense, parallel collagen fibers; 3. shorter, loosely arranged collagen fibers; 4. loose connective tissue and deep fibers of the coracohumeral ligament; and 5. the joint capsule [4]. The transition from the tendon to the bone tissue is also divided into four zones. The first zone, the tendon, is composed of parallel type I collagen (COL1) fibers and tenocytes. The second zone, the fibrocartilage, consists mainly of collagen type II (COL2) and type III (COL3). The third zone, the mineralized fibrocartilage, contains COL2, collagen type X and aggrecan. The fourth zone, the bone, consists of COL1 and minerals [5].

The supraspinatus tendon is composed mainly of parallel fibers of COL1, with smaller amounts of COL3 and COL2 [6]. The tenocytes, with a characteristic elongated, oval-, or spindle-shaped morphology, account for approximately 95% of the cellular population [7]. The extracellular matrix contains glycosaminoglycans (mainly hyaluronic acid), glycoproteins (mainly decorin, biglycan and aggrecan), proteoglycans, metalloproteinases (MMPs) and other smaller molecules [8]. Rupture of the supraspinatus tendon is associated with structural changes in the tendon tissue, especially at the torn end. Collagen fibrils and fibers become disorganized, and COL3 production increases [9]. Tenocytes exhibit heterogeneity in morphology and impaired function, and exogenous tenoblasts from the subacromial bursa and the synovial fluid migrate at the site of rupture [10]. The production of glycosaminoglycans and glycoproteins increases, and so does that of MMPs. Increased expression of metalloproteinase type 3 (MMP3) and type 13 (MMP13) has been found in cases of RC tear [11,12].

The subacromial bursa is a fluid-filled sac which reduces friction between the RC and the acromion and facilitates proper function of the supraspinatus muscle [13]. It is a highly vascularized and cellular tissue that contains mesenchymal stem cells, monocytes/macrophages, lymphocytes, growth factors and cytokines [14]. Rupture of the RC causes inflammation of the subacromial bursa and an increase in pre-inflammatory cytokines, including interleukin 1β (IL1β) and 6 (IL6) [15]. Its biological role in supraspinatus tendon tears includes: (1) the inflammatory response, through the proliferation of immune cells and the release of pro-inflammatory cytokines; (2) angiogenesis, both within the bursa and the tendon tissue; and (3) the proliferation and migration of mesenchymal cells into the tendon, with the aim of promoting healing and tissue regeneration [16].

Shoulder pain has a significant socioeconomic impact and is commonly related to rotator cuff tendinopathy and tear or subacromial bursitis [17,18]. In this setting, orthobiologics have been extensively used in recent years to accelerate the healing process during conservative treatment and to improve the outcomes of surgical management [19]. These treatment modalities can be divided into two main categories: cell-based therapies and autologous peripheral blood-derived products [20]. The first category includes autologous or allogenic mesenchymal stem cells and a stromal vascular fraction that contains a variety of regenerative cells, including stem cells [21]. The second category includes platelet-rich plasma, which is most frequently used, as well as a variety of enzymes and growth factors that can be isolated and applied, such as platelet lysate, autologous conditioned serum, gold-induced cytokines, growth factor concentrate, autologous protein solution and hyperacute serum [22].

Platelet-rich plasma (PRP) is an autologous blood-derived product that contains platelets and platelet-derived growth factors and cytokines [23]. Leukocyte-rich PRP (LR-PRP) contains also leukocytes, whereas leukocyte-poor PRP (LP-PRP) is pure without leukocytes [24]. The therapeutic effect of PRP has become increasingly popular for the treatment of various musculoskeletal pathologies, including RC tears, but the optimal platelet and leukocyte concentration is still debated [25]. Additionally, it has been applied as an adjuvant treatment to different orthopedic procedures, such as RC repair, due to its potential anti-inflammatory and regenerative effects [26].

Both clinical and laboratory studies have evaluated the effect of PRP on RC tendon tears [27,28,29]. Local injection of PRP at the site of RC tears has been associated with improved long-term outcomes, including reductions in pain and improvements in shoulder function [30]. Furthermore, the use of PRP as an adjunct during RC repair has been related to better clinical outcomes and lower re-tear rates [31,32]. In vitro studies have shown that PRP enhances cell proliferation, extracellular matrix gene expression and collagen synthesis in tenocytes obtained from patients with degenerative RC tendon tears [33,34].

According to our knowledge, the in vivo effect of PRP on subacromial bursa cell gene expression and the supraspinatus tendon morphology in the setting of RC rupture has not been investigated so far. Therefore, we conducted a randomized controlled trial in patients scheduled for arthroscopic RC repair comparing the microscopic and biologic effects of PRP injection with controls. We hypothesized that LP-PRP injection would be correlated with more well-organized tendon tissue and changes in gene expression from subacromial bursa cells.

## 2. Results

### 2.1. Demographics

Eight patients were enrolled in each group. Patient demographics are summarized in Table 1. The two groups did not differ with respect to age, gender distribution, affected side, smoking habit, comorbidities and classification of supraspinatus tendon rupture. In the PRP group, the injected PRP was analyzed, showing a mean platelet concentration of 773 ± 38 × 10^3^/μL and a mean leukocyte concentration of less than 200/μL.

### 2.2. Optical Microscopy Evaluation

**Figure 1:**
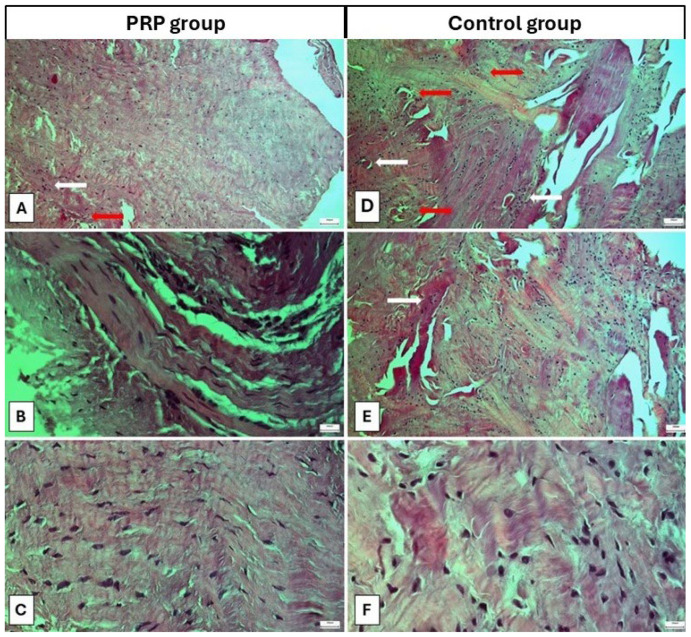
Optical microscopy of tendon samples. (**A**–**C**) Specimens from three different patients in the PRP group. (**D**–**F**) Specimens from three different patients in the control group. In the PRP group, oval- and spindle-shaped tenocytes were located within parallel, wavy collagen fibers. A few inflammatory cells were also present (white arrows), and blood vessels are indicated by the red arrows. In the control group, collagen fibers appeared more disoriented, showing both parallel and irregular distributions with a higher density of interposed oval- or round-shaped tenocytes. Inflammatory cells, which mainly accumulated around blood vessels (red arrows), are indicated by white arrows. Hematoxylin-and-eosin staining; magnification: ×100 (**A**,**D**,**E**) and ×400 (**B**,**C**,**F**); scale bar 200 pixels.

In the PRP group, abundant oval- and spindle-shaped tenocytes were identified between parallel collagen fibers with a nearly normal architecture. A few inflammatory cells were also found, indicating the absence of extended inflammation (Figure 1A–C). Conversely, in the control group, oval- and round-shaped tenocytes were lying within less organized collagen fibers that were disoriented. More extended regions with inflammatory cells were identified (Figure 1D–F).

The mean total modified Movin score in the PRP group was 6.5 ± 2.4 (range; 3–9), which was significantly lower than that of the control group (12.1 ± 2.9, range; 8–15) (*p* = 0.007). In the analysis of the different categories, the PRP group yielded a lower score than the control group in the categories “fiber structure” (1.5 ± 0.53 vs. 2.6 ± 0.5, *p* = 0.001), “fiber arrangement” (1.5 ± 0.53 vs. 2.6 ± 0.5, *p* = 0.001), “nuclear rounding” (1.8 ± 0.5 vs. 2.6 ± 0.5, *p* = 0.003), “inflammation” (0.6 ± 0.5 vs. 1.6 ± 0.5, *p* = 0.002) and “cell density” (0.6 ± 0.5 vs. 1.6 ± 0.5, *p* = 0.002). No difference was found in the category “angiogenesis” (0.5 ± 0.5 in the PRP group vs. 1.0 ± 0.8 in the control group, *p* = 0.149) (Table 2).

### 2.3. Electron Microscopy Evaluation

**Figure 2:**
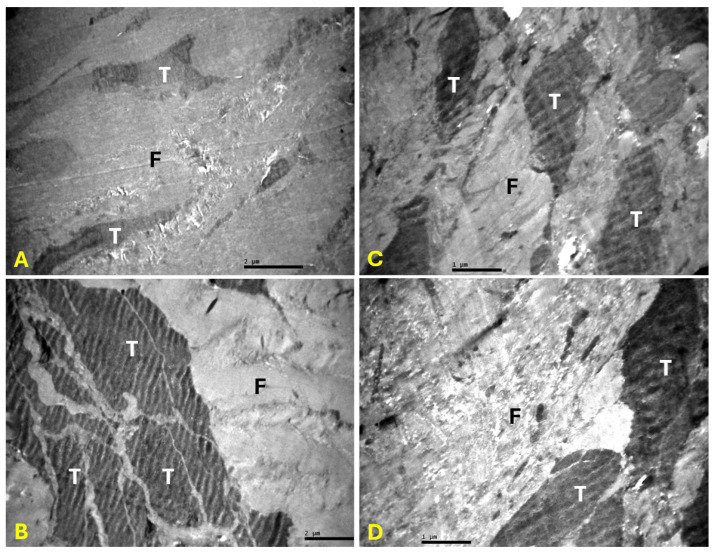
Electron microscopy of tendon samples from PRP group. Tenocytes (T) with multiple cytoplasmic processes were identified. Absence of disorientation of collagen fibers and parallel collagen fibers (F) between the tenocytes were mainly observed. Magnification: ×6000 (**A**,**B**), ×10,000 (**C**), and ×12,000 (**D**).

**Figure 3:**
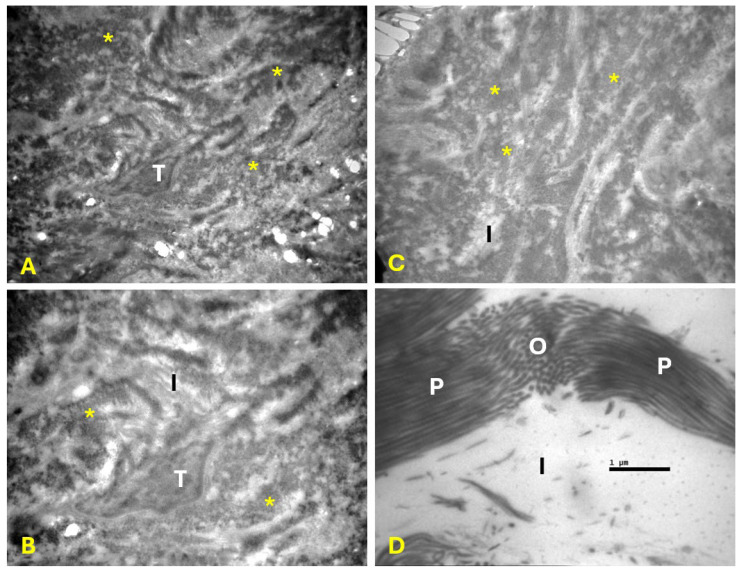
Electron microscopy of tendon samples from the control group. Disorientation of the collagen fibers (*) and disruption of the interstitial tissue (I) were dominant. Collagen fibrils appeared in a wavy, parallel (P) and oblique (O) orientation. Tenocytes (T) with short cytoplasmic processes were also identified between the collagen fibers. Magnification: ×6000 (**A**), ×10,000 (**B**,**C**), and ×30,000 (**D**).

In the PRP group, collagen fibers and fibrils were well organized. Tenocytes appeared to have multiple cytoplasmic processes (Figure 2). In the control group, collagen fibers and fibrils were disorganized with a disrupted architecture of the interstitial tissue. Tenocytes were identified with cytoplasmic processes that appeared shorter compared with those in the PRP group (Figure 3).

### 2.4. Gene Expression from Subacromial Bursa Cells

The results of the polymerase chain reaction (PCR) are presented in the Supplementary Materials, Table S1.

Seven out of eight samples from each group were evaluated for gene expression. The COL1, COL2, COL3 and MMP13 genes were investigated in seven samples from each group, the MMP3 and IL6 genes were investigated in six samples from each group. Finally, the IL1β gene was examined in six and four samples from the PRP and control groups, respectively.

**Figure 4:**
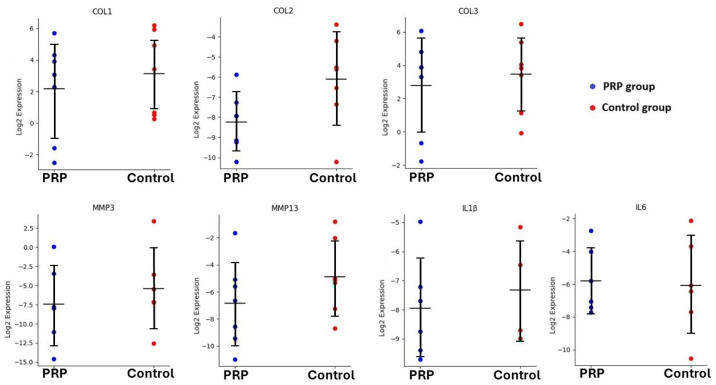
Relative gene expression in the PRP and control groups. Gene expression levels were normalized to β-actin and expressed as log_2_(2^−ΔCt^). Individual data points represent biological samples. Horizontal black lines indicate group means. Error bars show standard deviation.

The comparative analysis revealed no statistically significant differences between the PRP and control groups for any of the analyzed genes (all *p* > 0.05). However, the evaluation based on the predefined biological significance threshold identified that the COL2, MMP3 and MMP13 genes demonstrated lower expression in the PRP group compared to controls. More precisely, the fold change in the COL2, MMP3 and MMP13 genes was 0.23, 0.24 and 0.26, representing approximately 4.3-fold, 4.1-fold and 3.9-fold lower expression in the PRP group, respectively. These differences did not reach statistical significance but met the criterion for biological relevance. The discrepancy between statistical and biological significance likely reflects sample size limitations and inter-individual variability. In contrast, the COL1, COL3, IL1β and IL6 genes did not show any statistically or biologically significant differences between the two groups. Notably, the COL1 gene fold change was 0.51, close to the theshold of 0.50, which suggests biologically important downregulation in the PRP group (Table 3, Figure 4).

## 3. Discussion

The findings of this study indicate that PRP can affect the microscopic characteristics of the human supraspinatus tendon and the gene expression of subacromial bursa cells in the setting of degenerative RC tears in vivo. Following PRP injection, fewer areas of inflammation were identified, and the appearance of a supraspinatus tendon was closer to that of normal tendon tissue in terms of fiber structure and arrangement, nuclear rounding and cell density. Additionally, the gene expression in the subacromial bursa cells, including COL2, MMP3 and MMP13, was decreased after PRP injection.

The Movin score has been used to assess the morphological characteristics of tendon tissue [35]. Several modifications to this scoring system have been published [36,37,38]. In the current study, we used the Movin score modified by Chen, which is based on hematoxylin-and-eosin staining [39]. We found a lower total modified Movin score in the PRP group compared with controls. In the analysis of each category, lower scores for “fiber structure”, “fiber arrangement”, “nuclear rounding”, “inflammation” and “cell density” were noted in the PRP group. In the literature, PRP has also been correlated with a decreased Movin score when applied at the tendon rupture or repair site [40,41]. Yuskel et al. [40] injected PRP into repaired rat Achilles tendon after an induced rupture. Histological analysis on days 15 and 30 revealed lower Movin scores in the PRP group compared with controls at both time points. This finding was indicative of PRP’s potential to accelerate tendon healing. In a similar study, Genc et al. [41] found a lower Movin score on day 30 after PRP application to repaired Achilles tendon in a rat model compared with groups in which autologous conditioned serum or no adjunct were applied. The authors also reported increased COL1 production in the PRP group.

There is limited evidence for the effect of PRP on subacromial bursa cell biology. In an in vitro study, Hawthorne et al. [42] examined the effect of various biologic adjuvants used during RC repair on macrophage polarization in human supraspinatus tendon and subacromial bursa tissue. Compared to controls, PRP was associated with a decreased expression of interleukin-12 and tumor necrosis factor-alpha (TNF-α) by M1 proinflammatory macrophages and increased expression of interleukin-10, arginase and transforming growth factor-β by M2 anti-inflammatory macrophages. Furthermore, decreased expression of the M1 macrophage markers CD80, CD86, CD64 and CD16 and increased expression of the M2 macrophage markers CD163 and CD206 were observed in the PRP group. Finally, PRP was correlated with a decrease in nitric oxide production. In our study, we found that PRP reduced the gene expression of COL2, MMP3 and MMP13 in subacromial bursa cells by approximately 4.3-fold, 4.1-fold and 3.9-fold, respectively. Nonetheless, it remains unclear whether this downregulation contributes to improved tendon healing. Given that MMPs are enzymes responsible for collagen degradation, their reduced expression may enhance the potential for tendon repair; however, as they also play essential roles in matrix remodeling during the healing cascade, their blanket suppression may not be uniformly beneficial [43]. Furthermore, COL2 is mainly found at the fibrocartilage transition zone of tendon insertion [44]. Therefore, inhibition of its expression may negatively affect tendon–bone integration.

In the setting of RC tear, PRP has been associated with alternations in tenocyte gene expression and improved tendon-to-bone healing after repair [33,45]. Using supraspinatus tendon samples from patients with supraspinatus tendinopathy, Cross et al. [46] compared the effect of LR-PRP and LP-PRP on gene expression. In moderately degenerative tendons, the use of LP-PRP was related to increased expression of MMP type 9, IL-1β and the COL1:COL3 ratio, indicating promotion of normal collagen matrix synthesis and a decrease in cytokines associated with matrix degradation and inflammation. In another study, Jo et al. [47] noticed that the addition of calcium-activated PRP to cultured tenocytes derived from degenerated RC tissue increased the gene expression of total collagen, decorin, scleraxis and tenascin-T, thereby stimulating matrix synthesis. Moreover, PRP enhanced cell proliferation, suggesting that it can serve as a useful biological adjunct for regenerative healing of rotator cuff tears. Yoon et al. [48] investigated the effect of PRP on tenocytes derived from supraspinatus tendon tissue with or without a degenerative tear. Platelet-rich plasma promoted cell proliferation and matrix synthesis in both groups. The gene expression of decorin and tenascin-c and the overall synthesis of glycosaminoglycans was higher, whereas the COL3:COL1 ratio was lower, in tenocytes derived from degenerative tissue compared to those from normal tendon. In a laboratory study, Kelly et al. [49] cultured tenocytes derived from ovine infraspinatus tendons on six different media, one of which contained PRP. Higher production of COL1, which approached statistical significance, was noted in the PRP group, without any difference in the expression of IL-10 and TNF-α.

Symptomatic RC tears can be treated conservatively or surgically [50]. Conservative treatment includes per os medications, physical therapy and injection of steroids, PRP or stem cells [51,52]. Surgical options refer to RC repair of repairable tears and more advanced procedures, such as repair with graft augmentation, biodegradable subacromial spacer insertion, superior capsular reconstruction, tendon transfer and arthroplasty, for massive tears [53]. Evidence suggests that conservative management should be the first-line treatment of RC tears as, especially for partial-thickness and small full-thickness tears, it can provide similar outcomes to surgical repair [54,55,56,57].

Rotator cuff repair can be augmented with orthobiologics, including PRP, stem cells and biological scaffolds, that facilitate tendon-to-bone healing [58]. The use of these augmentation techniques has been associated with a reduced repair failure rate; however, their effect on functional outcome is debated [59]. In a recent meta-analysis of 13 randomized controlled trials, Gill et al. [60] concluded that the perioperative use of PRP during RC repair can reduce retear rate and improve pain in the short-term follow-up. In another meta-analysis, Vieira Ferreira et al. [61] did not find a significant benefit of RC repair augmentation with mesenchymal stem cells; however, they suggested a potential protective effect of stem cells against retear in the mid-term and long-term follow-up. In a systematic review of six comparative studies, Orozco et al. [62] found that although patch augmentation of RC repair reduced the retear rate, it did not improve the functional outcome. While the literature suggests that these biologic treatment options may improve the result of RC tendon repair, there is a need for comparative studies to evaluate whether one approach offers advantage over the others.

Subacromial bursitis has been related to subacromial impingement syndrome. The inflammation of the subacromial bursa causes pain that limits shoulder function [63]. First-line management includes physical therapy, anti-inflammatory medications and injections, while surgical treatment is reserved for refractory cases [64]. Physiotherapy alone can improve the symptoms, but its combination with corticosteroid injection seems to accelerate the beneficial effect [65]. Corticosteroid injections in the subacromial space, with or without ultrasound guidance, can reduce subacromial-bursitis-related pain and inflammation mainly in the short term [66]. In contrast, PRP injection seems to have a slower but longer-lasting effect on pain relief compared with corticosteroids [67].

This study should be considered on the subject of the following limitations. Firstly, the number of patients enrolled in each group was relatively small. The sample size provides adequate power only for large effects. Smaller effects—particularly for gene expression—may have been missed. Additionally, for IL-1β, the number of samples evaluated for the control group was reduced to four due to non-detectable expression; thus IL-1β findings are exploratory and should not be overinterpreted. Secondly, a ≥1 log_2_-unit threshold was used to identify potentially meaningful gene expression differences. Although this threshold has been used in the literature, there is no specific evidence supporting its biological relevance in rotator cuff pathology. Thirdly, obtaining biopsy specimens at the time of PRP injection might have provided a more accurate assessment of the effects of PRP on tendon and subacromial bursa biology. However, this would have required an additional operative procedure under regional or general anesthesia, which could also raise ethical concerns. Finally, we did not evaluate the effect of PRP on the clinical outcome of RC repair because of the small sample size. Such a comparison would require at least 40 patients in each group. Despite these limitations, to our knowledge, this is the first study to investigate the in vivo effect of PRP on the subacromial bursa cell gene expression and the supraspinatus tendon morphology in the setting of RC tear.

## 4. Materials and Methods

This randomized controlled trial has been registered in ClinicalTrials.gov with ID number NCT07456215. All described procedures were approved by the Ethics Committee of our hospital and our university (No. 15043—27/09/2023). All patients gave written informed consent to participate. The results of the first twenty patients enrolled in this randomized controlled trial have already been published [68]. This study presents the results of the next sixteen eligible patients.

### 4.1. Study Design

**Figure 5:**
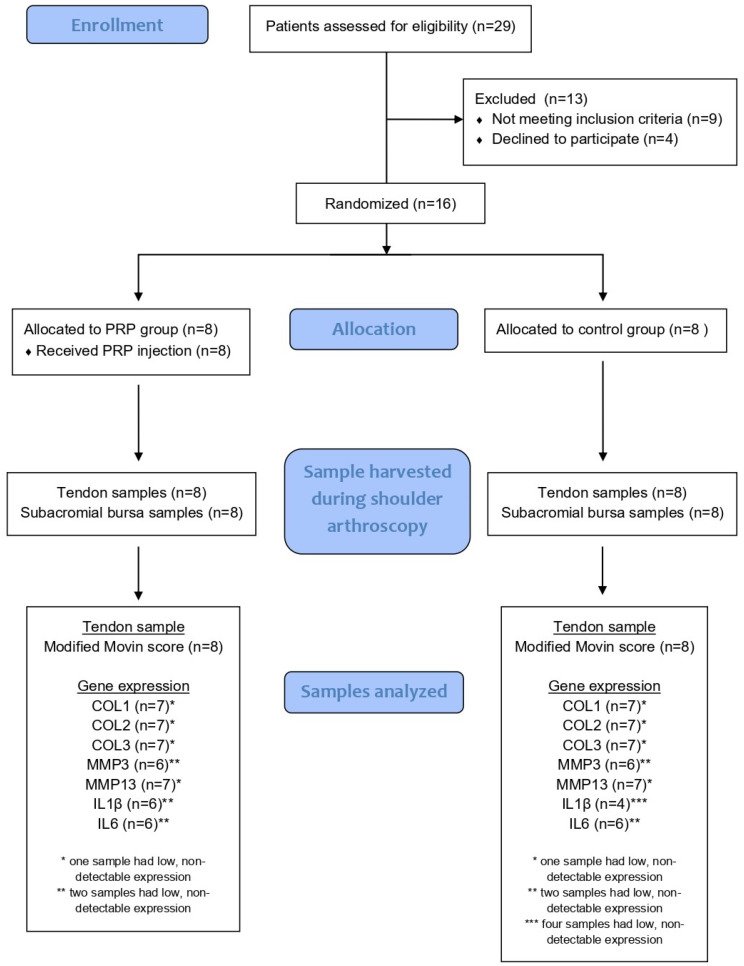
CONSORT flow diagram showing patient enrollment, allocation, sample collection and analysis.

This study presents a single-blinded randomized clinical trial. The randomization process involved alternating assignment of sixteen consecutive eligible patients into two groups: the experimental (PRP group) and the control groups. Eight patients were included in each group. Patients of the PRP group underwent an ultrasound-guided PRP injection in the subacromial space 6 weeks before the scheduled operation. In the control group, no injection was made prior to surgery. Subacromial bursa and supraspinatus tendon specimens from the lateral end of the torn tendon were harvested during shoulder arthroscopy. The subacromial bursa specimens were properly prepared for the measurement of specific gene expression, while the tendon specimens were examined under optical and electron microscopes. The pathologist inspected each sample blindly, and the results were collected, analyzed and presented. Patient enrollment, group allocation, sample harvesting, histological evaluation and gene expression analysis are summarized in the Consolidated Standards Of Reporting Trials (CONSORT) flow diagram (Figure 5).

### 4.2. Patient Selection

The study question was structured using the PICO model: (P) Population: patients with supraspinatus tendon tear confirmed on magnetic resonance imaging (MRI) who were to be treated with arthroscopic repair; (I) Intervention: injection of PRP 6 weeks prior to the scheduled operation; (C) Comparison: patients who did not receive PRP injection; (O) Outcome: modified Movin scores of supraspinatus tendon tissue and gene expression of subacromial bursa cells.

The following inclusion criteria were applied: (1) type 1 or 2, according to DeOrio and Cofield classification, full-thickness supraspinatus tears [69], (2) shoulder pain after failed conservative management of at least 3 months and (3) age between 40 and 65 years. Patients with grade 3 or 4, according to Fuchs classification [70], fatty infiltration of the supraspinatus muscle in MRI, history of injection around the shoulder joint during the past 12 months, history of former shoulder surgery or that were at high risk for surgery due to comorbidities were excluded from the study.

### 4.3. Preparation of PRP

The PRP was prepared using a TriCell PRP M Blood Separation Kit (Prosys, Tricell, Revmed, Seoul, Republic of Korea) according to the manufacturer’s instructions. Whole blood (27 mL) was taken from each patient in the PRP group and mixed with heparin sodium (5000 IU/mL) (3 mL). The mixture was centrifuged (Centrifuge 5420, Eppendorf AG, Hamburg, Germany) for 5 min at 3200 revolutions per minute until red blood cells were separated from plasma and buffy coat. The plasma and buffy coat were collected and were centrifuged again. Finally, 3–4 mL of LP-PRP, with 4 to 6 times greater platelet concentration compared to baseline levels and minimum number of leukocytes, was obtained.

### 4.4. Injection of PRP

In the study group, the PRP was injected into the subacromial space six weeks prior to scheduled arthroscopic surgery. The injection of PRP was administrated under ultrasound guidance (eZono^®^ 3000, eZono AG, Jena, Germany; linear transducer L3-12; 3–12 MHz) in a lateral-to-medial direction, just above the supraspinatus tendon.

### 4.5. Tendon and Subacromial Bursa Sample Harvesting

**Figure 6:**
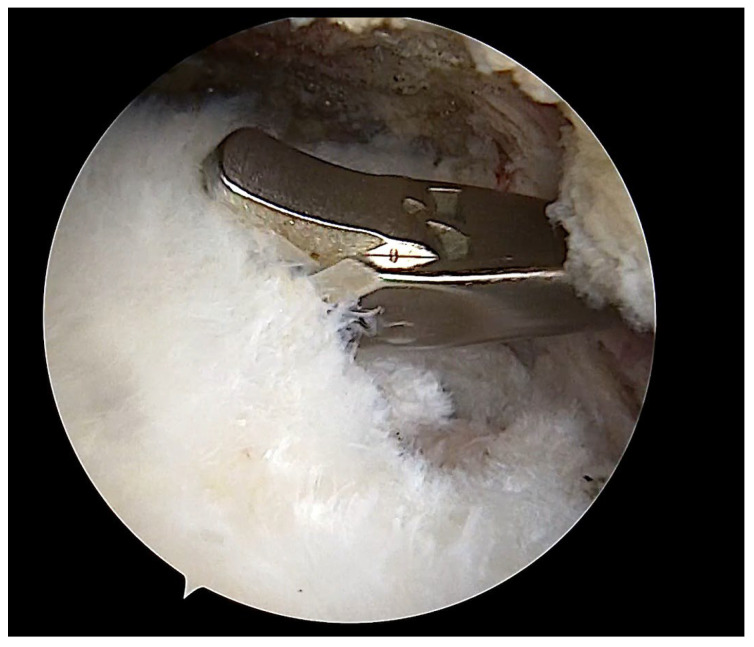
Arthroscopic image. The tendon specimen was harvested using a basket punch.

During the arthroscopic surgical procedure, a 3 × 5 mm full-thickness supraspinatus tendon specimen was harvested from the lateral edge of the tendon tear using a basket punch (Figure 6). The specimen was divided into two equal parts. One part, which was to be evaluated by optical microscopy, was embedded in sterile 10% formalin solution, and the second part, which was to be examined by electron microscopy, was sectioned into <0.5 cm^3^ pieces and placed in glutaraldehyde solution. The subacromial bursa specimen was harvested in a similar fashion and stored at −70 °C.

### 4.6. Tendon Sample Preparation for Optical Microcopy

The samples for the optical microscope examination were prepared with a method previously described [68]. Briefly, specimens underwent formalin fixation, graded alcohol dehydration, xylene clearing, and paraffin embedding. Paraffin blocks were sectioned at 3 μm thickness with eight sagittal sections per patient and mounted on slides, dried, deparaffinized, rehydrated, stained with hematoxylin and eosin, cleared in xylene, mounted with Canada balsam, and examined under light microscopy. A binocular light microscope (Olympus CX series biological microscope, Olympus Corporation, Tokyo, Japan) was used.

### 4.7. Tendon Sample Preparation for Electron Microcopy

Electron microscopy samples were prepared using a previously described protocol [68]. Specimens were fixed in 3% glutaraldehyde for 2 h and post-fixed in 1% osmium tetroxide for 1 h. They were then stained with 1% uranyl acetate for 16 h, dehydrated through ascending ethanol concentrations, and embedded in Epon resin. Ultrathin sections (50–100 nm) were cut and stained with Reynolds’ stain before analysis. The microscopy was performed using a TEM JEOL 1011 electron microscope at 80 kV (JEOL-Tokyo, Tokyo, Japan).

### 4.8. Assessment of Tendon Sample

In the optical microscopy scanning, the histopathological assessment was based on the modified Movin score [39]. This score assesses six parameters: fiber arrangement, fiber structure, rounding of the nuclei, inflammation, angiogenesis and cell density (Table 4). Each variable is quantified in a scale between 0 and 3, with 0 = normal, 1 = slightly abnormal, 2 = abnormal and 3 = markedly abnormal. Two histopathologists independently assessed the samples and were blinded to group allocation, and disagreements were resolved by a third histopathologist.

A descriptive approach was used to compare electron microscopy findings between the two groups.

### 4.9. Assessment of Gene Expression

To assess the gene expression of the subacromial bursa cells, reverse transcription polymerase chain reaction (PCR) was utilized. This process included two steps. During the first step and after warming up the specimen, specific primers were used to identify the RNA sequences of the studied genes. Reverse transcriptase was applied to synthesize complementary DNA (cDNA) from RNA. In the second step, the cDNA was multiplied, and gene expression was quantified by performing quantitative real-time PCR (qRT-PCR).

The expression of the following genes was investigated: *COL1*, *COL2*, *COL3*, *MMP3*, *MMP13*, *IL-1β* and *IL-6*. The qRT-PCR primer sequences are depicted in Table 5. For the collagen, the primer for the α1 chain of each type was used (COL1A1, COL2A1 and COL3A1). The gene expression of β-actin was used to normalize the results.

Non-detectable qRT-PCR values occurred for some genes due to low expression (below detection) and were considered missing data and excluded from that gene’s analysis; therefore, sample size varied by gene and is reported for each comparison.

### 4.10. Sample Size

The primary endpoint was the total modified Movin score. A two-group comparison (PRP vs. control) was carried out using a two-sided α = 0.05. With the available recruitment capacity, the study was powered to detect large effects; specifically, *n* = 8 per group provides ~80% power to detect a standardized mean difference of approximately d ≈ 1.5. Therefore, the trial was designed to detect a large histological effect, and the gene expression analyses were considered exploratory.

### 4.11. Statistical Analysis

All the extracted data were transcribed into SPSS (IBMCorp. Released 2017. IBM SPSS Statistics for Windows, Version 25.0. Armonk, NY, USA: IBM Corp.) and subsequently analyzed. Normality was assessed using the Shapiro–Wilk test. Because the total modified Movin scores deviated from normality in both groups, between-group comparisons were performed using the Mann–Whitney U test (two-sided α = 0.05). Additionally, Welch’s *t*-test was also conducted as a parametric sensitivity analysis. Similarly, for the comparison of gene expression, Welch’s *t*-test was used due to small or unequal sample sizes and Mann–Whitney U tests were conducted as non-parametric sensitivity analyses. All tests were two-sided, and statistical significance was assumed at a *p* value of <0.05.

Relative gene expression was calculated using the comparative Ct (ΔCt) method, where ΔCt was defined as ΔCt = Ctgene − Ctβ-actin. Positive differences indicate increased expression and negative differences indicate decreased expression in the PRP group relative to controls. Relative expression levels were expressed as 2^−ΔCt^. For statistical analysis, values were log_2_-transformed (−ΔCt), which corresponds directly to log_2_ fold changes in gene expression.

For the comparison of gene expression, in addition to statistical testing, a biological significance threshold was predefined. A difference of ≥1 log_2_ unit between groups, corresponding to a ≥2-fold change in gene expression, was considered biologically meaningful, independent of statistical significance. This means that “fold-change” values of >2 or <0.5 indicated increased or decreased gene expression in the PRP group, respectively. This approach was used to identify genes exhibiting potentially relevant expression changes in the context of a limited sample size.

## 5. Conclusions

The subacromial injection of LP-PRP in patients with degenerative supraspinatus tendon tears was associated with improved tendon histological appearance, as indicated by the lower modified Movin score, and with a biologically relevant decrease in COL2, MMP3 and MMP13 gene expression from subacromial bursa cells, although no statistically significant gene expression difference was found. These findings suggest that PRP may influence both tendon microscopic architecture and the biological activity of the subacromial bursa in vivo; however, the clinical significance of these molecular changes and their impact on the tendon-to-bone healing process and postoperative outcomes remain uncertain. Larger randomized studies including advanced imaging and functional evaluation and longer follow-up are required to clarify the mechanisms of PRP action and determine whether its effect on histology and gene expression can be correlated with meaningful improvements in the rotator cuff healing process and patient recovery.

## Figures and Tables

**Table 1:** Patients’ demographic data.

Parameter	PRP Group (8 Patients)	Control Group (8 Patients)	*p* Value
Mean age (years) (SD)	58.3 ± 4.9	57.5 ± 5	0.767
Gender (male/female)	2/6	1/7	0.522
Side (dominant/nondominant)	6/2	6/2	1.000
Smoking	3	1	0.248
Comorbidities (hypertension, diabetes, thyroidopathy, dyslipidemia)	2	2	1.000
Classification of rupture	3 small/5 medium	4 small/4 medium	0.614

**Table 2:** The modified Movin scores of the included patients.

Patient	Group	Fiber Structure	Fiber Arrangement	Nuclear Rounding	Inflammation	Angiogenesis	Cell Density	Total
1	PRP	2	2	2	1	1	1	9
2	PRP	1	1	1	1	0	0	4
3	PRP	1	1	1	0	0	0	3
4	PRP	1	1	2	0	0	1	5
5	PRP	2	2	2	1	1	1	9
6	PRP	2	2	2	1	1	1	9
7	PRP	2	2	2	1	1	1	9
8	PRP	1	1	2	0	0	0	4
1	Control	3	3	3	2	1	2	14
2	Control	2	2	2	1	0	1	8
3	Control	2	2	2	1	1	1	9
4	Control	3	3	3	2	2	2	15
5	Control	3	3	3	2	1	2	14
6	Control	2	2	2	1	0	1	8
7	Control	3	3	3	2	2	2	15
8	Control	3	3	3	2	1	2	14

**Table 3:** Comparison of gene expression between PRP and control groups.

Gene	Group	NoP	Mean (log_2_) *	SD (log_2_)	Δlog_2_ (PRP–Ctr) **	*p*-Value	Fold Change (PRP/Ctr)	95% CI of Fold Change	≥1 log_2_ Unit
*COL1*	PRP	7	2.17	3.08	−0.96	0.542	0.51×	0.05–5.21	No
	Control	7	3.13	2.63					
*COL2*	PRP	7	−8.24	1.44	−2.12	0.061	0.23×	0.05–1.09	Yes
	Control	7	−6.12	2.25					
*COL3*	PRP	7	2.79	2.9	−0.67	0.641	0.63×	0.08–5.24	No
	Control	7	3.46	2.28					
*MMP3*	PRP	6	−7.46	5.23	−2.04	0.516	0.24×	0.002–26.10	Yes
	Control	6	−5.43	5.25					
*MMP13*	PRP	7	−6.85	3.13	−1.96	0.236	0.26×	0.02–2.76	Yes
	Control	7	−4.89	2.74					
*IL1* *β*	PRP	6	−7.96	1.75	−0.63	0.605	0.65×	0.09–4.52	No
	Control	4	−7.32	1.84					
*IL6*	PRP	6	−5.8	2.02	0.30	0.843	1.23×	0.12–12.28	No
	Control	6	−6.09	2.96					

Abbreviations: CI: confidence interval; COL: collagen; Ctr: control group; IL: interleukin; MMP: metalloproteinase; NoP: number of patients; PRP: PRP group. * Gene expression was normalized to β-actin and analyzed on a log_2_(2^−ΔCt^) scale. ** Difference in mean normalized gene expresion values between the two groups.

**Table 4:** The modified Movin score [39].

Variables	Grade 0	Grade 1	Grade 2	Grade 3
Fiber structure	Continue, long fiber	Slightly fragmented	Moderately fragmented	Severely fragmented
Fiber arrangement	Compacted and parallel	Slightly loose and wavy	Moderately loose, wavy and cross to each other	No identifiable pattern
Rounding of the nuclei	Long, spindle-shaped cells	Slight rounding	Moderate rounding	Severe rounding
Inflammation	<10%	10–20%	20–30%	>30%
Angiogenesis	<10%	10–20%	20–30%	>30%
Cell density	Normal pattern	Slight increase	Moderate increase	Severe increase

**Table 5:** Quantitative real-time PCR primer sequences.

Gene	Accession Number	Forward Primer	Reverse Primer
*COL1A1*	NM_000088.3	TGACCTCAAGATGTGCCACT	ACCAGACATGCCTCTTGTCC
*COL2A1*	NM_033150.2	CGCACCTGCAGAGACCTGAA	TCTTCTTGGGAACGTTTGCTGG
*COL3A1*	NM_000090.3	GCTGGCATCAAAGGACATCG	TGTTACCTCGAGGCCCTGGT
*MMP3*	NM_002422.3	TGGGCCAGGGATTAATGGAG	GGCCAATTTCATGAGCAGCA
*MMP13*	NM_002427.3	CCTTCCCAGTGGTGGTGATG	CGGAGCCTCTCAGTCATGGA
*IL-1β*	NM_000576	TCCAGGAGAATGACCTGAGC	GTGATCGTACAGGTGCATCG
*IL-6*	NM_000600	TGAGGAGACTTGCCTGGTGA	TTGGGTCAGGGGTGGTTATT

## Data Availability

The original contributions presented in this study are included in the article/Supplementary Material. Further inquiries can be directed to the corresponding author.

## Supplementary Materials

The following supporting information can be downloaded at: https://www.mdpi.com/article/10.3390/ijms27073002/s1.
