# Supplementary material for: The Biological Effect of Platelet-Rich Plasma on Subacromial Bursa and Torn Supraspinatus Tendon: A Randomized Controlled Trial
Source: Int J Mol Sci. 2026 Mar 26;27(7):3002. doi: 10.3390/ijms27073002 (PMC13073171; doi:10.3390/ijms27073002)
Supplement: Supplementary file 1 [file ijms-27-03002-s001.zip › ijms-4159109-supplementary.pdf]

Supplementary Table S1. The results of gene expression using real-time PCR.

**Patient**	**Group**	**β-actin**	**Col1A1**	**Col2A1**	**Col3A1**	**MMP3**	**MMP-13**	**IL-1β**	**IL-6**
**1**	PRP	26,255	20,555	32,140	20,170	34,025	27,890	31,230	ND
**2**	PRP	23,445	19,120	32,585	18,620	31,410	30,080	33,150	27,475
**3**	PRP	24,090	21,020	31,360	20,200	35,170	29,190	31,305	31,520
**4**	PRP	21,285	18,995	31,505	17,970	ND	26,880	28,980	24,020
**5**	PRP	20,090	16,185	28,040	16,205	34,660	28,655	ND	27,145
**6**	PRP	24,495	27,010	32,425	26,280	27,940	33,945	33,890	32,215
**7**	PRP	22,980	24,550	32,230	23,655	22,920	33,955	31,730	28,780
**8**	Control	27,815	21,620	33,325	24,395	33,305	29,820	32,970	34,260
**9**	Control	26,995	22,075	30,375	21,615	ND	32,320	ND	ND
**10**	Control	23,365	19,950	30,725	19,545	30,575	28,375	29,820	27,050
**11**	Control	25,975	20,045	30,170	19,485	22,580	26,795	ND	36,505
**12**	Control	22,195	21,510	32,405	18,165	34,735	30,875	30,900	29,880
**13**	Control	26,615	26,355	32,230	25,470	30,200	33,860	35,595	32,695
**14**	Control	26,305	25,770	32,840	26,370	33,435	31,440	ND	28,445

Abbreviations: ND: not detectable
